# Accuracy of the detection of binding events using 3D single particle tracking

**DOI:** 10.1186/s13628-017-0035-8

**Published:** 2017-03-23

**Authors:** Sara Carozza, Jamie Culkin, John van Noort

**Affiliations:** Huygens-Kamerlingh Onnes Laboratory, Leiden University, Postbus 9504, Leiden, 2300RA Netherlands

**Keywords:** Single particle tracking, Diffusion, Binding event, Mean square displacement analysis

## Abstract

**Background:**

Nanoparticles can be used as markers to track the position of biomolecules, such as single proteins, inside living cells. The activity of a protein can sometimes be inferred from changes in the mobility of the attached particle. Mean Square Displacement analysis is the most common method to obtain mobility information from trajectories of tracked particles, such as the diffusion coefficient *D*. However, the precision of *D* sets a limit to discriminate changes in mobility caused by biological events from changes that reflect the stochasticity inherent to diffusion. This issue is of particular importance in an experiment aiming to quantify dynamic processes.

**Results:**

Here, we present simulations and 3D tracking experiments with Gold Nanorods freely diffusing in glycerol solution to establish the best analysis parameters to extract the diffusion coefficient. We applied this knowledge to the detection of a temporary change in diffusion, as it can occur due to the transient binding of a particle to an immobile structure within the cell, and tested its dependence on the magnitude of the change in diffusion and duration of this event.

**Conclusions:**

The simulations show that the spatial accuracy of particle tracking generally does not limit the detection of short binding events. Careful analysis of the magnitude of the change in diffusion and the number of frames per binding event is required for accurate quantification of such events.

**Electronic supplementary material:**

The online version of this article (doi:10.1186/s13628-017-0035-8) contains supplementary material, which is available to authorized users.

## Background

### Introduction

Cells present a dynamic environment for the biomolecules that orchestrate life: important processes such as intracellular or intramembrane trafficking [1–3] protein dynamics [4, 5] and gene delivery [6, 7] can be studied in detail by analyzing the mobility of the molecules involved. Single-Molecule Tracking (SMT) is a powerful tool to investigate such dynamic processes. SMT discloses information unobtainable using ensemble techniques, because following molecules individually can reveal variations in behavior that occur during the process, including rare events that are otherwise obscured in the ensemble. The high precision of SMT relies on the possibility to localize a single molecule with higher accuracy than the diffraction limit [8]. Ultimately, the accuracy of localization depends on the optical brightness of the molecule. Because most biomolecules can not be detected using optical microscopy, they need to be labeled with fluorescent markers like organic dyes or fluorescent proteins. Alternatively, metal or semiconductor nanoparticles have been used as labels to track single molecules. Single-Particle Tracking (SPT) [9] is advantageous over SMT because nanoparticles are generally brighter than fluorophores and can therefore be tracked with better precision. Moreover, as opposed to single fluorophores, nanoparticles don’t bleach, which extends the time span over which a single molecule can be followed. However, nanoparticles are larger than single fluorophores, and will thus affect the mobility of the molecules of interest.

From SPT one can obtain long time traces of single molecules, that are then analyzed to quantify mobility. The Mean Square Displacement (MSD) of the particle reveals characteristic modes of mobility like free diffusion, confined diffusion and active transport, which are characterized by parameters such as diffusion coefficient (*D*), velocity and confinement size. The ability to track individual molecules, labeled with nanoparticles, with nanometer precision and over long times would make it possible to observe transient changes in the mobility of the molecule that could not be observed using other methods. For example, the binding of a transcription factor to its DNA target has been challenging to detect at the single-molecule level. Though Fluorescence Correlation Spectroscopy (FCS) and SMT approaches have been used to study this process [10, 11], the short length of the traces, due to photobleaching and/or diffusion out of the detection volume, generally directs data analysis to ensemble properties rather than those of single molecules. Therefore, SPT could provide a unique alternative for monitoring the dynamics of an attached molecule.

How reliable are the mobility parameters extracted from such SPT experiment? In the case of active transport, the localization accuracy is the most important factor influencing the precision of the particle velocity. In the case of diffusion an evaluation of the accuracy of *D* is more complex: diffusion is a stochastic process, and this requires the measurement of many independent localizations to obtain *D* with high precision. The precision of *D* is of high relevance for biological experiments, as it sets a threshold to discriminate a biologically meaningful change in diffusion from the intrinsically stochastic variations.

Here we investigate how accurate the diffusion coefficient of a particle can be measured in a SPT experiment, and how well we can detect a transition in its diffusion behavior. The issue of accuracy of diffusion coefficients has been addressed before, with a theoretical approach and simulations [12, 13], but mainly in 2D. 2D SPT can provide higher temporal resolution, but the images are limited in space to single planes and the tracking can be performed only as long as the particle stays in the plane: the use of 2D SPT is therefore limited to tracking in cell compartments that can be approximated to 2D such as the cell membrane [14, 15]. The simulations in this report extend such analysis to 3D tracking experiments.

We used Gold Nanorods (GNRs) as labels for 3D SP using Two Photon excitation. GNRs are cylinder-shaped gold nanoparticles with sizes ranging between few tens to several hundreds of nanometers: they are bigger than organic dyes and fluorescent proteins, and can therefore slow down the tracked molecule. Nevertheless they offer several advantages over fluorophores: their luminescence is up to 100 times higher (yielding a spatial resolution that is 10 times better, [16]), and can be tracked for hours as they are not affected by bleaching or blinking [17]. Moreover, GNRs are easy to functionalize and are compatible with live cells conditions [18].

We acquired multiple z sections forming a 3D image stack using a multifocus two photon microscope as described before [16]. Two photon excitation reduces out-of-focus excitation and yields a good spatial resolution both in the longitudinal and axial directions. We scan an array of focal spots using a scanning mirror: this way we obtain a wide-field illumination of the sample.

Some 3D SPT techniques have a higher temporal resolution compared to z sectioning, like for example the use of cylindrical lens to extract 3D positioning [1]. However, the use of astigmatism is not compatible with two-photon excitation, and thus lacks the benefits of higher signal-to-noise; Total Internal Reflection microscopy [19] gives high spatial and temporal resolution, but within a limited 3D area, not sufficient to cover the entire volume of a cell; orbital tracking [20] tracks only one particle at the time and cannot benefit from the high throughput of parallel tracking. A good alternative to multi-focus excitation two-photon microscopy is two-photon Light Sheet Microscopy [21] that provides good penetration depth in the sample and a comparable acquisition speed; for SPT these two techniques present similar challenges.

The outline of this paper is as follows: first, we address the influence of positional accuracy of the 3D tracking scheme on the precision of the extracted MSD with simulations; then we analyze the accuracy of the obtained diffusion coefficient with simulations and experiments; we optimize the parameters that are used to obtain *D* from the MSD; finally we simulate traces containing a change in diffusion behavior and establish the experimental boundaries for resolving such changes.

## Methods

### Experimental setup

The acquisition of 3D movies of single GNRs was performed on a home-built two-photon multifocus scanning microscope as previously reported in [16], with some small changes. A near IR pulsed laser (Coherent Chameleon Ultra) was used for excitation; the laser beam was split in an array of 625 beams by a diffractive optical element (DOE, custom made by Holoeye). A fast scanning mirror, driven with an Archimedean spiral function, was used to scan the array of beams over the sample: this way we obtained a wide and homogenous excitation on an area of about 60 *μ*m x 60 *μ*m, and collect images of tens of GNRs within this area. A piezo-stage (PIfoc, PI) was used to move the objective in the z-axis to collect 3D images. We acquired images with an EMCCD Camera (Photometrics QuantEM 512SC). The frame size was 400 pixels x 400 pixels, corresponding to 70 *μ*m x 70 *μ*m and the separation between z slices was typically 0.5 *μ*m. We acquired 10 z slices per stack, at a rate of 10 frames/s: the time resolution of our 3D localization was therefore 1 s/stack.

### Sample preparation

Samples of GNRs of two different sizes were used: 47 ±4 nm x 14 ±2 nm GNRs were synthesized through a seed-mediated method [22], while 53 ±6 nm x 16 ±3 nm GNRs were purchased from Nanopartz (A12-25-780-CTAB). Both GNRs samples were functionalized with a polyethylene glycol (PEG) layer before use. GNR sizes were obtained from Transmission Electron Microscope (TEM, JEOL JEM 1010) images of both batches. The TEM images also provided a measure for the size dispersion within the two samples. The GNR sizes used for our theoretical calculations were increased by the thickness of a PEG layer. The size of the PEG layer (which cannot be seen in TEM) was measured independently using Fluorescence Correlation Spectroscopy (FCS, [23]), yielding an effective PEG layer thickness of 8.1 nm (see Additional file 1: Figure S1). GNRs were first suspended in small volumes of demineralized water, then glycerol was added to reach the desired concentration of 95 and 90% glycerol. For SPT in glycerol both GNR samples were excited at a wavelength of 770 nm.

### Simulations

Simulations of movies of diffusing GNRs were performed in LabVIEW using the following procedure: a set of 3D trajectories was created, according to a given diffusion coefficient *D* (or multiple values of *D*, in case of changes in behavior); a stack of empty frames was then filled with a 3D Gaussian peak for each time coordinate, and amplitude and standard deviation of the peak were set using typical values obtained experimentally for single GNRs (amplitude=1000 a.u., *s*_*xy*_=300 nm, *s*_*z*_=650 nm); Poissonian noise was added to each pixel in the peak in order to simulate shot-noise; an offset (1000 a.u.) and a background noise (1 a.u.) were added to the entire 3D stack of images, reflecting the camera gain settings and detection noise. As opposed to experimental movies, in simulated movies we introduced only one GNR to prevent incorrect trajectory assignments when GNRs would cross. We simulated videos with a frame rate of 10 frames/s, as typically collected by our setup. The frame size was 300 pixels x 300 pixels (corresponding to about 52 *μ*m x 52 *μ*m, and the separation between z slices was 1 *μ*m.

### Data analysis

**Fig. 1:**
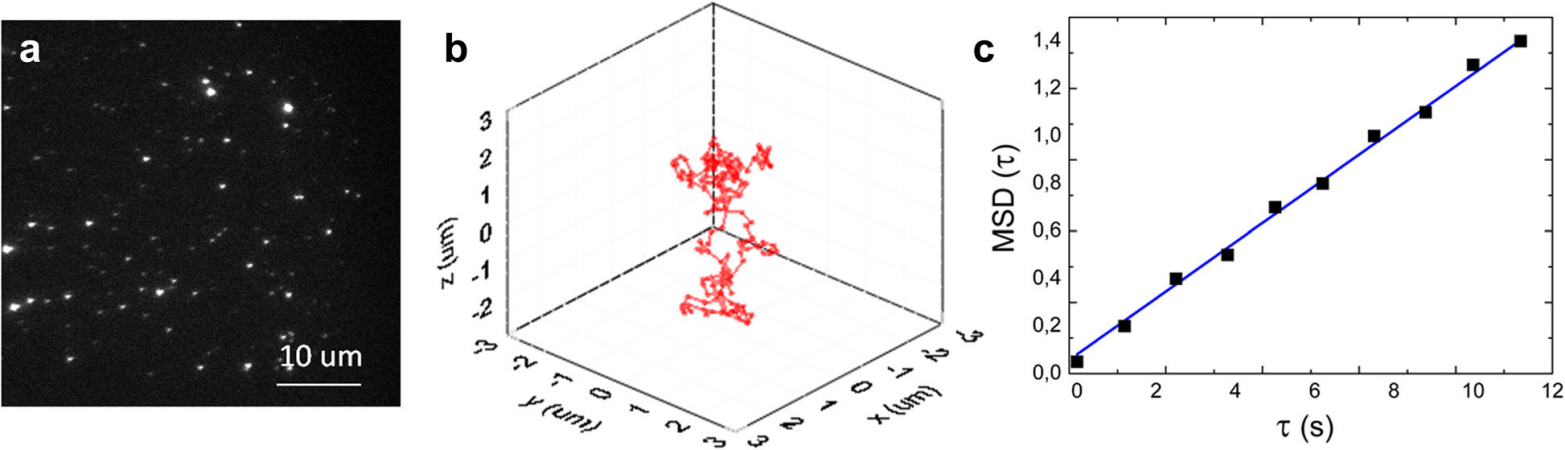
Steps to extract the diffusion constant of single GNRs in glycerol. From a movie of 3D stacks of frames (as the frame in **a**), trajectories of single GNRs were extracted (**b**), and on each of them a Mean Square Displacement (MSD) analysis was performed. The MSD plot (**c**) was fitted to a line with a slope that corresponds to the diffusion coefficient, and offset proportional to the 3D positional accuracy

Image analysis was also performed in LabVIEW. The same analysis was applied to simulated and real movies. In each 3D stack of images, peaks were detected and fitted with a 3D Gaussian function: from the fit we obtained position, intensity, offset and width of each peak. When more than one trace was present in the movie, peaks were connected to traces using a minimal excursion criterion. Once traces were obtained, an MSD analysis was performed. An illustration of the method is shown in Fig. 1, and details of the MSD analysis process are described in the next section.

## Theory

### Localization accuracy

Figure 1 a shows a typical 2D image of a number of GNRs, whose peaks are convoluted with the Point Spread Functions (PSFs) of the microscope. The localization uncertainty *σ* of a single particle in a 2D fluorescent image follows [8, 24]:

1 $$ \sigma = \sqrt{\frac{s^{2}}{{N}_{p}}+\frac{a^{2}}{12N_{p}}+\frac{8\uppi s^{2} b^{2}}{a^{2} {N}_{p}^{2}}}  $$

where *s* is the width of the PSF, *N*_*p*_ is the number of photons, *a* is the pixel size and *b* the number of photons in the background noise. As is characteristic for shot-noise, the uncertainty in position decreases with increasing number of photons. The uncertainty in the case of 3D images will depend on the 3D image acquisition scheme. Previously, we reported an experimental increase in x and y accuracy in 3D data that originated from the additional photons recorded in all frames above and below focus that contribute to a 3D peak ([16]). These measurements were made using fixed, immobile GNRs. In the results section we will quantify this effect. However, changes in positions between slices in a stack will affect the positional accuracy.

### Accuracy of MSD analysis

For now we will ignore the movement between slices in the stack and analyze single traces (Fig. 1 b) by calculation of the Mean Square Displacement. The MSD of a trajectory is the average of all the squared displacements *r* ^2^ occurring within time steps of different duration *τ*:

2 $$ MSD(\tau) = \frac{1}{{n}_{\tau}}\sum_{i=1}^{{n}_{\tau}}\left({r_{i+\tau}-r_{i}} \right)^{2}  $$

where *n*_*τ*_ is the number of steps, equal to (*T*- *τ*)/ *τ*. *T* is the total length of the trace and *τ* is the time lag between displacements. The diffusion of a particle is quantified by the coefficient *D*, described by the Stokes-Einstein equation:

3 $$ D = \frac{\mathrm{k}T}{6 \uppi \upeta R}  $$

where k is the Boltzmann constant, *T* the temperature, *R* the radius of the particle and *η* the viscosity of the medium. For free diffusion in an isotropic medium the MSD has a linear dependence on *τ* [4], and in 3D it results in:

4 $$ MSD(\tau) = 6D\tau + 6 \sigma^{2}  $$

Fitting Eq. 4, one can obtain the diffusion coefficient *D*, as well as the 3D localization accuracy *σ*. Figure 1 c shows an example of an MSD plot. A parameter that has a large influence on the accuracy of the fit is the number of MSD points that are included in the fit.In the example in Fig. 1 c, the GNR trace is about 100 points long, and we fitted the first 10 MSD points to obtain *D*. When dealing with shorter traces though, the points in the MSD plot at larger time delays become increasingly random, due to the stochastic nature of diffusion and the fewer measurements that contribute to the mean. Including these points in the fit may yield an erroneous D. Due to this inherent statistical variance in the MSD, the error on the obtained *D* can be significant and will depend on the number of points that are included in the fit. The relative error in *D* is defined as:

5 $$ \rho = \left| \frac{{D}-{D}_{\text{measured}}}{{D}}\right|  $$

Qian et al. [13] showed that *ρ* depends on the total length of the trajectory *N* and on the number of fitting points *n*, and approximates to:

6 $$ \rho = \sqrt{\frac{2n}{3K}}  $$

where *K*=*N*-*n*. Weighting MSD points according to the sample size could yield a better accuracy, but Thompson [8] showed that the effect of this correction is negligible. Michalet [12] extended Quian’s analysis to conditions with a finite localization uncertainty to determine the best number of fitting points for the analysis. He calculates the relative error to be:

7 $$ \begin{aligned} \rho &= \left\{{\vphantom{\frac{1}{{K}}}}\frac{{n}}{6K^{2}}\left(4Kn^{2}+2K+{n}-{n}^{3}\right)\right.\\&\quad\left.+\frac{1}{{K}} \left[2nx + x^{2} \left(1+\frac{1-\frac{{n}}{{K}}}{2}\right)\right] \right\}^{1/2} \end{aligned}  $$

where *x* is the reduced positional uncertainty and is defined as:

8 $$ x = \frac{\sigma}{{D}\Delta t}  $$

and *Δ* *t* is the sample time. Eq. 7 converges to Eq. 6 for *x* = 0, large *N* (*N* ≈ 1000) and *K*>>*n* [12]. In our work we use Michalet’s formula for *ρ* as we have a non-zero positional uncertainty and traces shorter than 1000 points. Michalet showed that choosing a non-optimal number of fitting points results in a diffusion coefficient noticeably larger than the actual one. He calculated the best number of fitting points to be:

9 $$ {n} = 2+2.3 x^{0.52}  $$

Therefore, the optimal number of fitting points to use depends on positional uncertainty, diffusion coefficient and sampling time. In a real experiments the expected *D* is typically not known, so *n* is not easy to evaluate. An estimate of the order of magnitude of the *D* to expect is a first good step. A higher sampling rate or a lower precision increases the value of the optimal *n* to use. We tested Michalet’s results with 3D simulations using different positional uncertainties and diffusion coefficients. Then we validated these results with experiments using GNRs with known *D*, compared *D* to the value measured with SPT and calculated the relative error *ρ*.

### Detection of changes in *D*

**Fig. 2:**
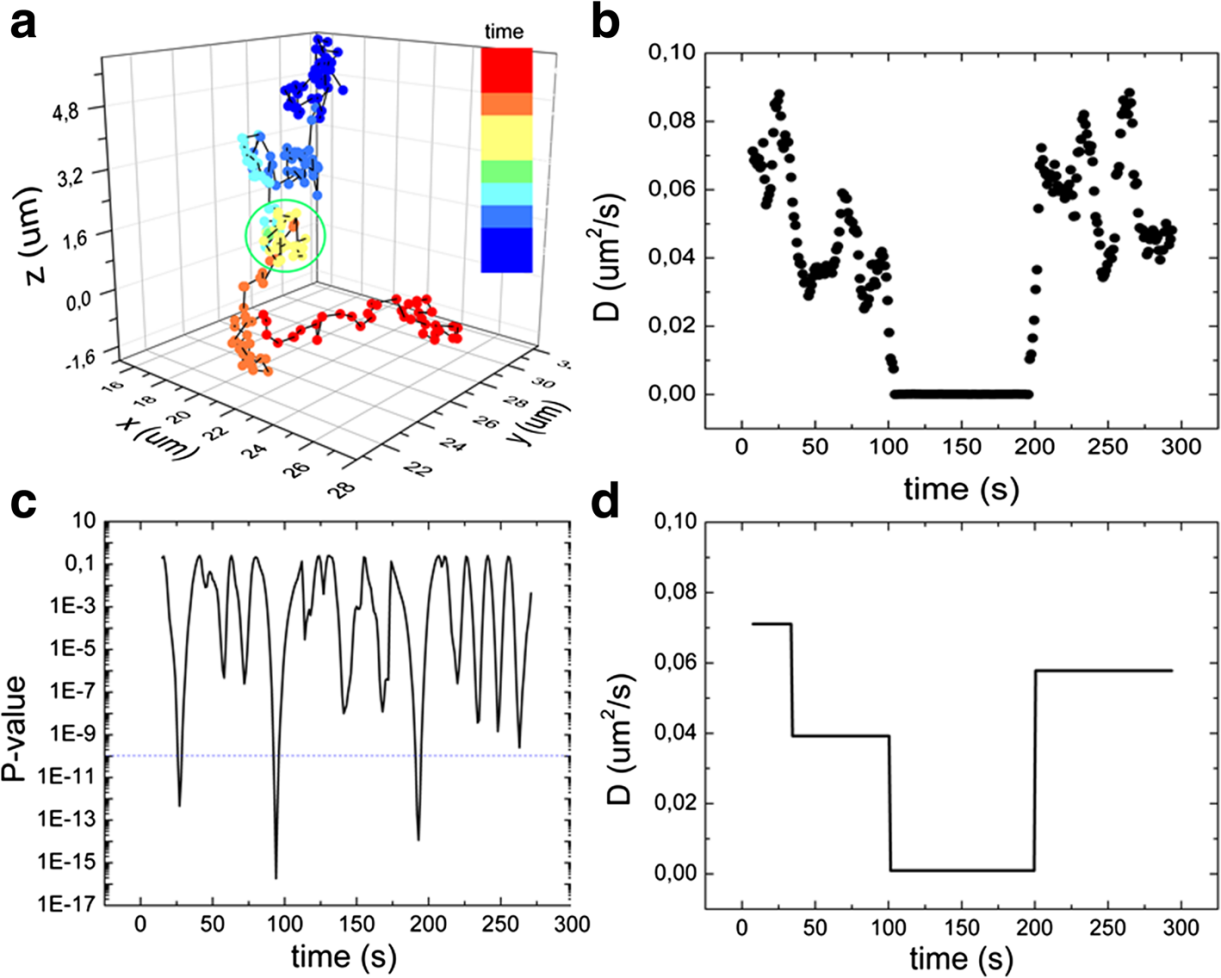
Detection of diffusion transitions. **a** A simulated trajectory, which contains a brief period of reduced mobility. In this gap *D* was 0,0001 *μ*m^2^/s, while the *D* in the rest of the trace was 0,05 *μ*m^2^/s. The gap is highlighted with a green circle. **b** The diffusion coefficient along the trajectory *D*(t) was calculated using a 15 points rolling-window method. **c** A Welch test yielded minima in the *P*-value plot, that indicate possible transition points used to divide the trajectory in subtraces. **d** MSD analysis of each subtrace yielded *D* values as shown

Having a well-defined, constant *D*, is however highly simplistic when doing SPT in cells: over time a molecule will undergo transitions in the diffusion behavior. An example of these traces is depicted in Fig. 2 a. We simulated and analyzed traces containing a transition in diffusion, in particular a period with a lower diffusion coefficient (Fig. 2 a), mimicking for example the binding of a particle to a fixed structure in the cell. To analyze these traces, we needed to detect the transition points. From a rolling window MSD analysis, a plot of the variations of *D* within the trace was obtained (Fig. 2 b). This *D*(t) plot was then analyzed with a Student’s T-test, evaluating the probability that two populations belong to the same distribution. In our case we used a modified version of Student’s T-test, the Welch’s test [25], optimized for populations with different variances. It calculates the T-statistic as:

10 $$ T = \frac{X_{1}-X_{2}}{\sqrt{\frac{s_{1}^{2}}{{N}_{1}^{2}}+\frac{s_{2}^{2}}{{N}_{2}^{2}}}}  $$

where *X*_1_,*X*_2_ are the means of the two samples, *s*_1_,*s*_2_ their variances and *N*_1_,*N*_2_ the samples sizes. The probability that the two samples are described by the same distribution is calculated using the T-distribution probability density function [25]:

11 $$ p(T, \nu) = \frac{\Gamma \left(\frac{\nu + 1}{2}\right)}{\sqrt{\uppi \nu} \Gamma \left(\frac{\nu}{2}\right)} {\left(1+\frac{T^{2}}{\nu} \right)}^{-\frac{\nu + 1}{2}}  $$

in which *Γ* is the gamma function.

The degrees of freedom *ν* are approximated by the Welch-Satterthwaite equation as:

12 $$ \nu \approx \frac{{\frac{s_{1}^{2}}{{N}_{1}^{2}}+\frac{s_{2}^{2}}{{N}_{2}^{2}}}^{2}}{\frac{s_{1}^{4}}{{N}_{1} \left({N}_{1}-1\right)}+\frac{s_{2}^{4}}{{N}_{2} \left({N}_{2}-1\right)}}  $$

A *p*-value is calculated for each point in the *D* plot, considering two windows of the same size around the point. The minima in the probability plot (Fig. 2 c) correspond to the points in the trace where a diffusion transition is most likely to happen. Rolling windows with different sizes (*N*_1_ and *N*_2_ in Eqs. 10 and 12) didn’t show noticeable differences. We chose a rolling window size of 15 steps, and a Welch test sample size of 15 or 10, when the gap was shorter than 15 steps. Transition points were assigned using a threshold for P and the initial trace was divided in subtraces. We tested different values for the threshold, and we obtained the best compromise between false negative and false positive results with a value of 10^-10^.

As shown in Fig. 2 c, not all the detected transition points corresponded to real transitions: some were misassigned due to stochastic fluctuations in *D*. We performed a second Welch test on these subtraces using window sizes corresponding to the entire subtraces length. The transitions confirmed by the second test were accepted as real transition points. Despite this second statistical test, it was not always possible to assign each transition point correctly. For example in Fig. 2 c at *t* = 25s a change in *D* was wrongfully detected. A new MSD analysis was finally done on the final subtraces to obtain the mean *D*, which is plotted in Fig. 2 d.

## Results and discussion

### Spatial and temporal resolution

**Fig. 3:**
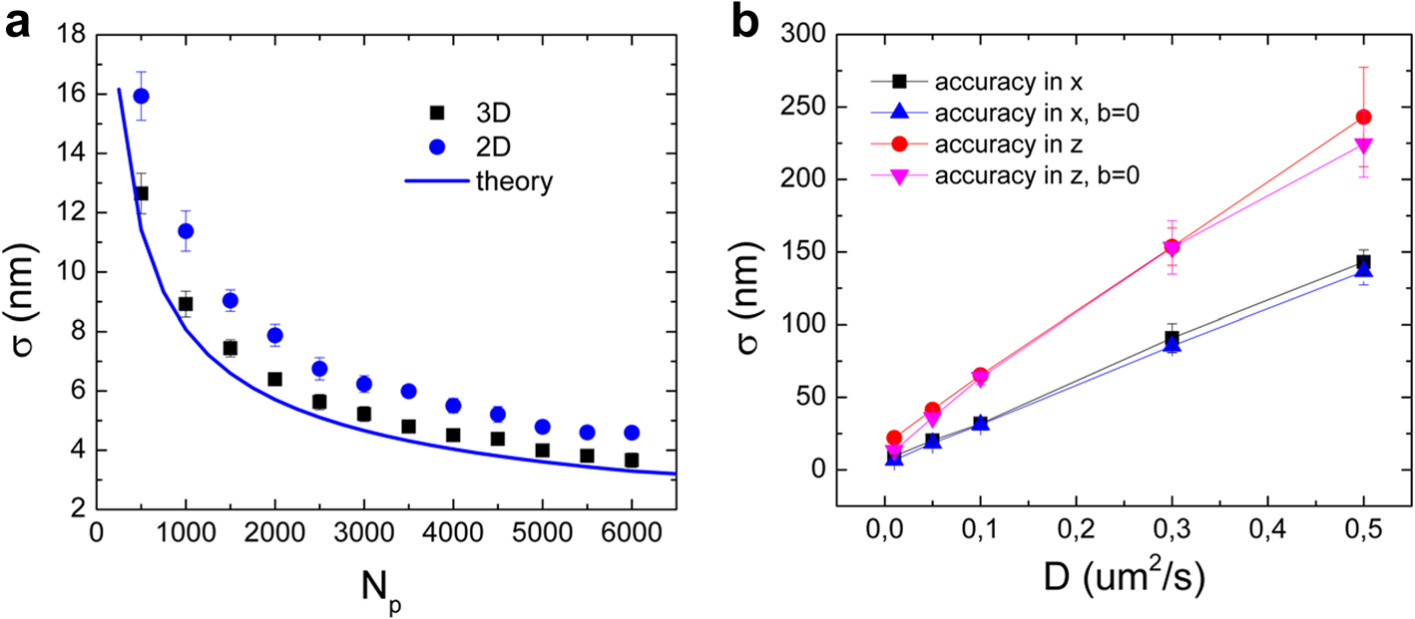
The positional uncertainty in 2D and 3D simulations depends on the brightness and the temporal resolution. **a** The localization uncertainty improves with increasing number of photons emitted from the GNR. The *blue line* represents the theoretical value obtained from Eq. 1. The images obtained from simulations were analyzed in 2D (fitting only one slice per 3D stack, *blue dots*) and in 3D (fitting the whole 3D stack, *black squares*). In 3D, the localization accuracy increases as compared to the 2D case. Each point in the graph is an average of 10 sets of 200 simulated images. The background noise was kept constant at *b* = 0.5. **b** The effect of the movement of the particle between frames for an acquisition time of 10 fr/s. As expected, the uncertainty increased with the diffusion coefficient

We first performed simulations to obtain the positional accuracy for 3D images with fixed peak positions. We tested cases with different numbers of photons *N* at fixed background noise b. We simulated static GNRs: the uncertainty was calculated from the difference between the input coordinates and the coordinates obtained from the Gaussian fit and plotted in Fig. 3 a. For 2-dimensional data only the central frame in each 3D stack was used. In this case, the positional uncertainty was consistently worse than expected based on Eq. 1. A similar discrepancy between theory and simulations was reported previously [8], and explained with the approximations used to derive Eq. 1. As anticipated, using 3D images decreases the positional uncertainty, due to the larger number of photons collected for a peak.

In the analysis of dynamic data, the temporal resolution plays an important role: the finite time between acquisitions can obscure fast dynamic processes. Moreover, in real experiments, the movement of the particle occurs also between slices within a 3D stack: we simulated this movement within a stack for a range of diffusion coefficients: as shown in Fig. 3 b, the effect of the movement within stacks can be dramatic for large diffusion coefficients. The positional uncertainty in the x-y plane for the lowest diffusion constant (*D* = 0.01 *μ*m^2^/s) is about 9.5 ± 0.6 nm, for the highest (*D* = 0.5 *μ*m^2^/s) *σ* is 143.0 ± 8.4 nm. In the z direction, the uncertainty follows the same trend but is even more pronounced. In the experiments on GNRs performed with our setup, the number of photons collected was very high, due to the high brightness of the two-photon signal of GNRs and low background. From Eq. 1 we calculate a positional accuracy of 4 nm (see Additional file 1: Figure S2) for an average *N*_*p*_ of 4000 photons. However, due to GNR movement between slices, the positional uncertainty is increased: considering the diffusion coefficient range expected for our experiments (between 0.02 and 0.07 *μ*m^2^/s), we expect the effective positional uncertainty in x,y to be around 20 nm, and in z around 40 nm. The uncertainty values obtained from the MSD fit is arund 40 nm: this value includes the x-y and the z components, and is comparable to the *σ* value in z obtained from simulations.

### Factors that determine the uncertainty in the detection of *D*

**Fig. 4:**
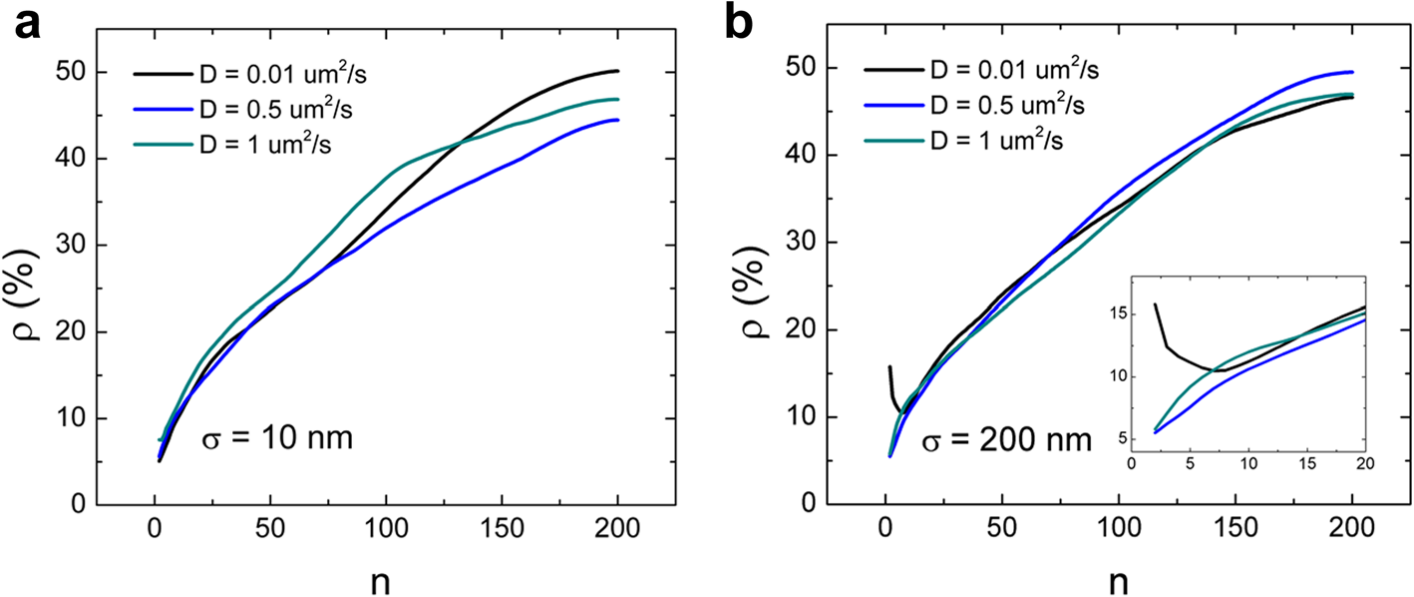
Stochastic variations in diffusion limit the accuracy of *D* measurements. **a** In case of low positional uncertainty (*σ*= 10 nm), the best number of fitting points is 2, for different values of *D*. **b** In case of higher positional uncertainty (*σ* = 200 nm), errors in position detection dominate the error in *D* for small displacements, and the best number of MSD points increases (see inset). Each point in the plot is the average *ρ* obtained from 100 simulations of 200 points traces

**Fig. 5:**
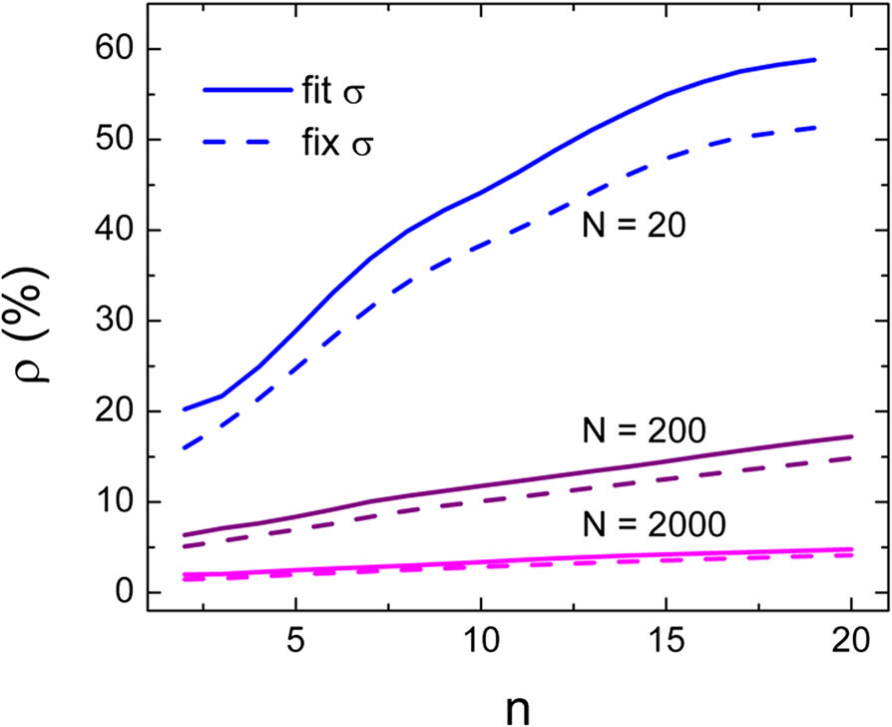
Optimizing the accuracy of *D* measurements for single trajectories. Using longer traces improves the estimate of *D*, lowering *ρ*. Fixing the positional uncertainty *σ* (*dashed line*) in the MSD fit reduces in the relative error compared to fitting it (*continuous line*). Each point is the average *ρ* obtained from 100 simulated traces with length as indicated in the legend and *D* = 1 *μ*m^2^/s

The stochastic nature of diffusion is another source of uncertainty in the determination of the diffusion coefficient *D*. Following Eq. 6, the length of the trace and the number of MSD fitting points have a large influence on the error in *D*. In Fig. 4 a, results from simulations show that the best number of fitting points for data with low positional uncertainty is 2, for different values of *D*, in accordance with Michalet’s results. When the positional uncertainty increases (Fig. 4 b), it has a large influence on the first MSD points, so more MSD points are required for an accurate determination of *D*. The length of the analyzed traces also affects the precision of the obtained *D* (Fig. 5): longer traces allow for a better statistics in the calculation of the MSD. The positional uncertainty can be calculated from the measurement independently using the number of photons (Eq. 1). Figure 5 shows that fixing the positional uncertainty *σ* in the MSD fit slightly improves the final result.

In summary, these precautions can reduce the error on the obtained *D*: using long traces, fixing the positional uncertainty of the MSD fit, and limiting the fit to the first two MSD points. Nevertheless, even with high positional accuracy, one will obtain relatively large errors in *D* when measuring for finite times due to the stochastic nature of diffusion.

### Experimental validation of *D* accuracy using GNRs in glycerol

**Fig. 6:**
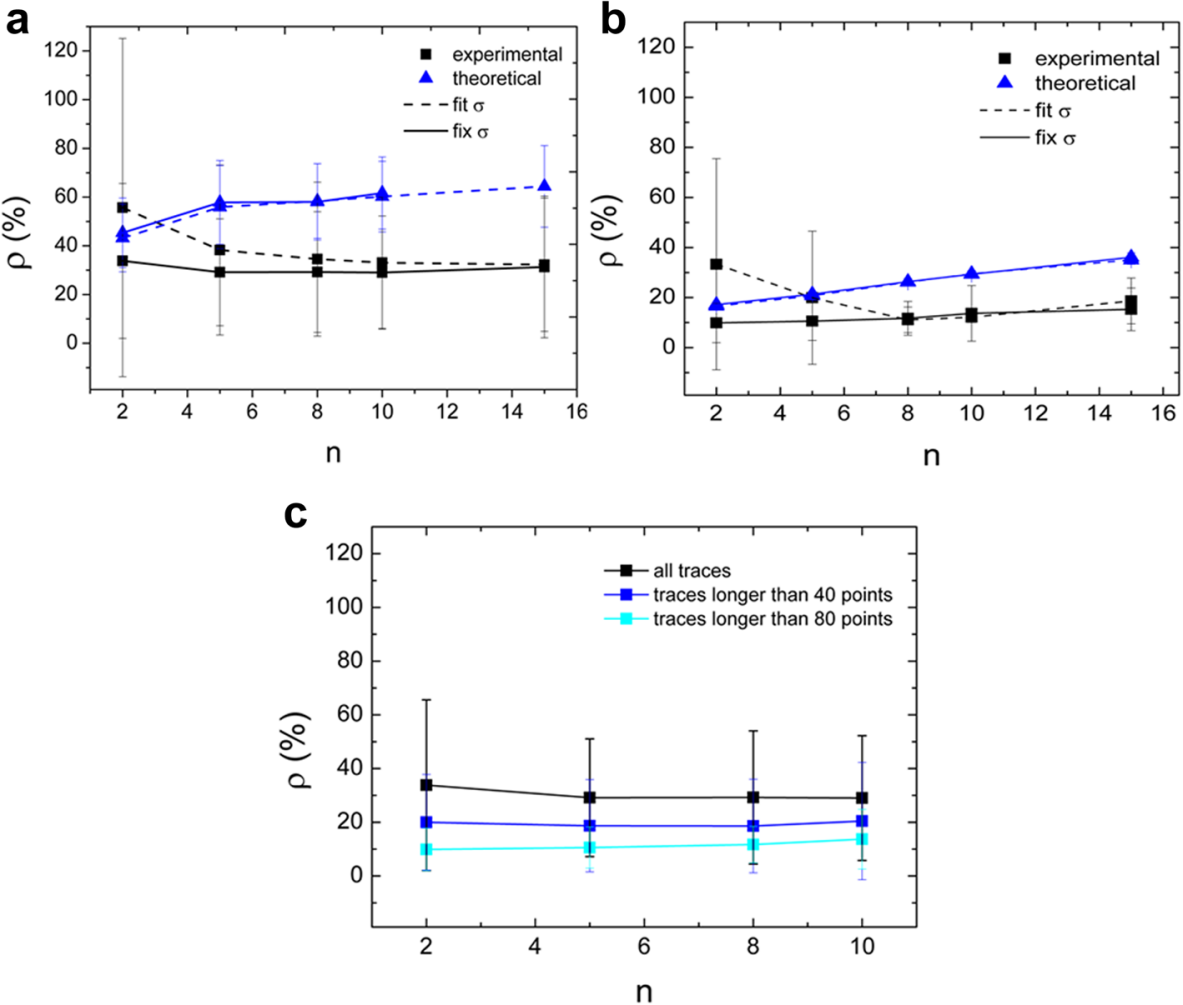
Optimal number of MSD points. Simulations and experimental data show that two points of the MSD plot are optimal for accurate fitting of *D*. The squares represent experimental values of *ρ*, calculated using Eq 5. The data were obtained from traces of 53 nm x 16 nm GNRs in 95% glycerol. The *triangles* represent the theoretical values of *ρ*, obtained with Eq 7. In **a**), traces of all lengths were considered (between 10 and 100 points), while in **b**) only traces longer than 80 points (about 2 min) were considered. In both conditions there is not much difference using different numbers of fitting points. In **c**) we compare the values of *ρ*(*D*) obtained from traces of all lengths (from 10 to about 100 points, *black squares*) with the ones obtained from only traces longer than 40 points (*blue squares*) and 80 points (*cyan*); in all three cases the analysis was performed fixing the positional uncertainty

We next tested our results on experimental traces of GNRs diffusing in glycerol with a well-known diffusion coefficient, rather than in a cellular environment, which is not homogeneous and therefore the diffusion coefficient would not be well-defined. We compared the statistical variations in *D* to the variations predicted based on the size dispersion of our GNR samples. Experiments were performed with two GNRs sizes and two glycerol concentrations. The expected values of *D*, calculated using Eq. 3, are listed in Additional file 1: Table S1. The values of *D* are at least two orders of magnitude smaller than the typical diffusion coefficients of proteins, due to the large size of the GNRs. Smaller GNRs may be used, but this would imply a weaker luminescence and a faster diffusion, which make it more difficult to accurately quantify the mobility. What follows is the results obtained from the first sample (52 nm x 16 nm GNRs in 95% glycerol), while the results from the other samples are summarized in Additional file 1: Table S1. In Fig. 6 a, the relative errors *ρ* obtained experimentally are compared to the theoretical errors (Eq. 7). Fixing *σ* lowers the error in the estimate of *D*, especially when a smaller number of MSD points is used. The collected traces had a large variation in length: as the GNRs were free to move, the trace length was limited to the time the GNRs stayed in the volume of view. Consistent with Eqs. 6 and 7 and the simulations, longer traces feature a more accurate *D*. In Fig. 6 b only the traces longer than 80 points were used for analysis: this decreased the relative error from 40% to less than 20%. Curiously, while the theoretical value of the relative error increases with the number of MSD fitting points, in the experimental values it had little or no influence. Using only the long traces, the precision slightly decreased with a number of MSD points larger than 5. In all cases the errors on *D* obtained experimentally were smaller than the ones calculated theoretically: this is not surprising, as the theoretical errors correspond to the standard deviation of the MSD curve, hence to the maximum value of the error [12, 13]).

In Fig. 6 c, we compare the experimental values (obtained fixing the positional uncertainty) for all the traces, traces longer than 40 points (about 1 min) and 80 points (about 2 min). The first thing to notice is the dramatic decrease in the *D* error when using longer traces. For the longest traces, the smallest number of MSD fitting points yields the smallest error. Therefore, the minimum error in the calculation of *D* is obtained using only traces longer than 2 min, fixing the positional uncertainty and using only 2 fitting points: in these conditions we got a relative error as low as 10%.

**Fig. 7:**
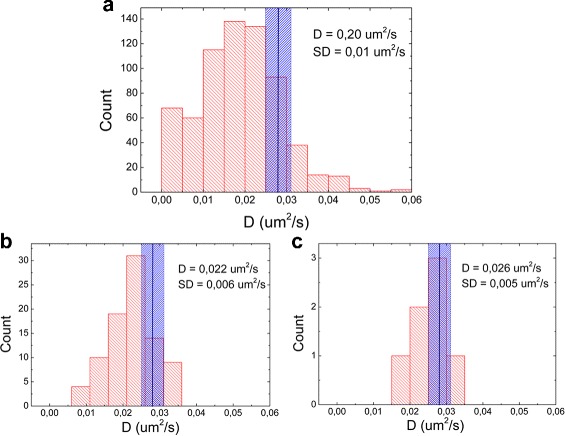
The diffusion constant measured using single particle trajectories is underestimated when using short traces. The distribution of the measured *D* is obtained using all traces (**a**) or only traces longer than 40 points (**b**) or 80 points (**c**) for 53 nm x 16 nm GNRs in 95% glycerol. The center of the *blue bar* represents the expected value (calculated from Eq. 3); its width follows from the size dispersion measured from TEM images. The experimental *D* values are somewhat smaller than the expected ones and this difference, as well as the standard deviation, decrease for longer traces

In Fig. 7 a the measured values of *D* are compared with the expected ones (calculated with Eq. 3). The variation in *D* based on the size dispersion of the GNRs, measured in TEM images, is depicted in the histograms using a blue shade around the expected value. As seen before, longer trace lengths improve the accuracy of *D*: when we limited the analysis to traces longer than 40 points (about 1 minute, Fig. 7 b) and 80 points (about 2 min, Fig. 7 c) the measured *D* increases from 0.020 *μ*m^2^/s to 0.022 *μ*m^2^/s and 0.026 *μ*m^2^/s, where the expected *D* was 0.028 *μ*m^2^/s. The relative errors in *D* obtained for this GNR sample and other samples are reported in Additional file 1: Table S1. In the experiments with shorter GNRs, the relative errors were higher, due to their faster diffusion which results in shorter traces. The variation in the measured *D* was always larger than the variation predicted based on the size dispersion (reported in the same table), because of the stochastic variations in *D* that increase its variability.

### Detection of changes in diffusion in single particle trajectories

One of the unique possibilities of SPT is to follow a single molecule over a long time, and to directly detect changes in its behavior. The previous discussion on the difficulties to obtain a correct *D* implies, however, major challenges. In this paragraph we tested how accurately a temporary reduction in diffusion constant of a particle (a ’gap’) can be detected. Following the approach above, MSD analysis was performed with 2 points and fixed positional accuracy calculated from the intensity of the peak. The diffusion coefficient used for the initial and final phases was 0.05 *μ*m^2^/s, which we typically measure for GNRs inside cells (both in nucleus and cytoplasm). We varied *D* in the gap from 0.0001 *μ*m^2^/s to 0.035 *μ*m^2^/s. The residual mobility of a protein bound to DNA has been reported to be in this range [26, 27]. The initial and final phases were 100 s, while we tested different lengths of the gap phase. We evaluated the effectiveness of the detection in the obtained diffusion coefficient and gap duration (*D*_*gap*_ and *t*_*gap*_) in every set of 100 simulations. We considered *D*_*gap*_ correct when it was within ± 20% of the set *D*, and we considered *t*_*gap*_ correct when it was within ± 10% of the set length. An example of a trace simulated using a *D*_*gap*_ of 0.0001 *μ*m^2^/s is shown in Fig. 2 a. In this figure, the gap is not clearly seen in the trajectory (as the time points are very close to each other), but it is easily distinguishable in the *D* plot. In real experiments the difference in mobility can be smaller.

**Fig. 8:**
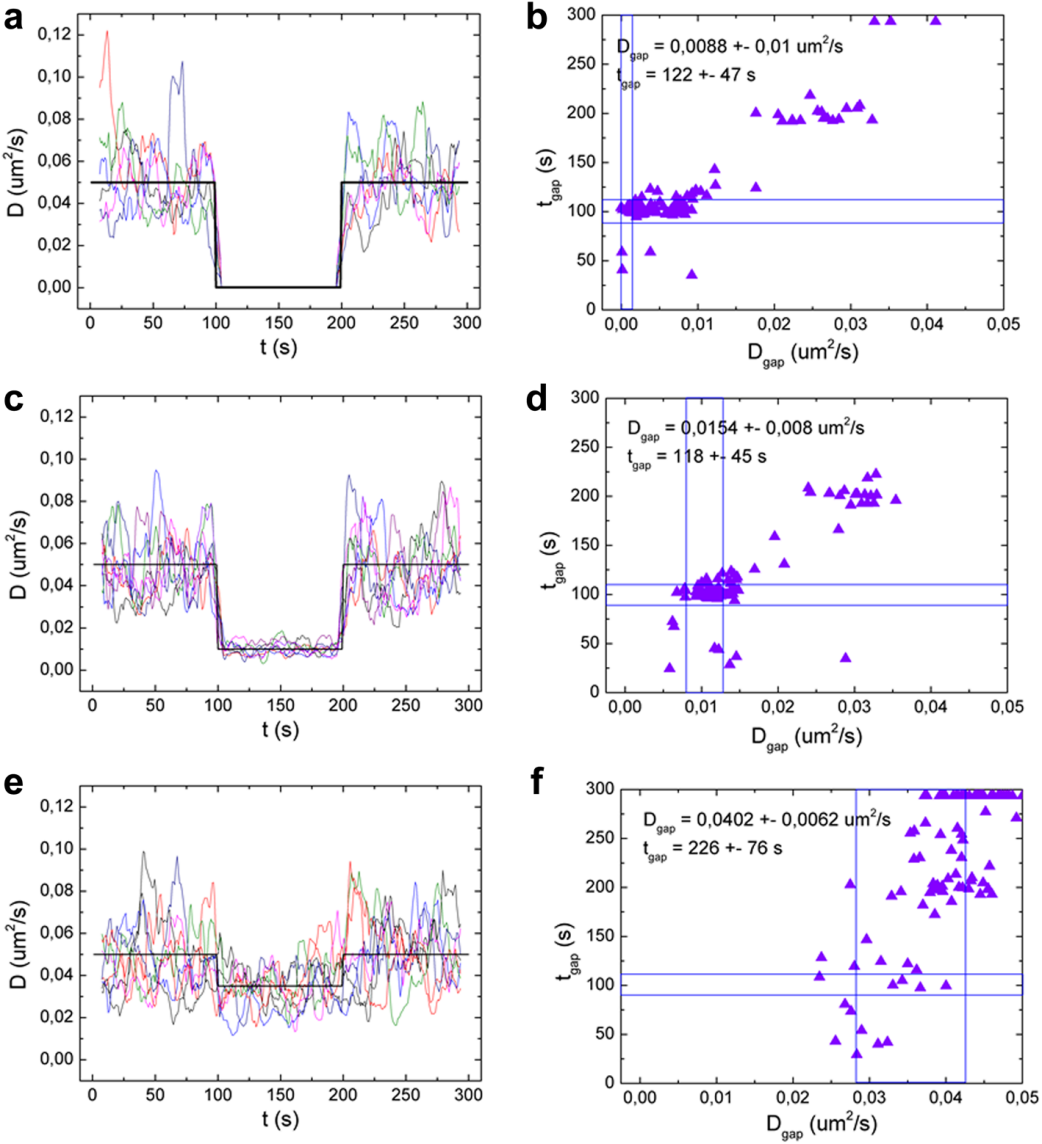
Limits to the detection of changes in diffusion. *D(t)* plots obtained from the rolling-window analysis (**a**, **c**, **e**) and scatter plots of *D*_*gap*_ vs the length of the gap (**b**, **d**, **f**). The simulated *D*(t) is plotted in *black line*. The *D* outside the gap was set to 0,05 *μ*m^2^/s. *D*_*gap*_ = 0.0001 *μ*m^2^/s for **a**, **b**, *D*_*gap*_= 0,01 *μ*m^2^/s for **c**, **d** and *D*_*gap*_=0,035*μ*m^2^/s for **e**, **f**. For every case, 100 traces were simulated and analyzed: **a**, **c** and **e** show the *D*(t) plot for 8 example traces. Results from all 100 traces are shown in **b**, **d** and **f**. The ranges of correct *D*_*gap*_ and *t*_*gap*_ are highlighted with *blue lines* in the scatter plot. The traces were analyzed using a rolling window and a Welch sample of 15 points

First, we simulated traces with different *D*_*gap*_, keeping the gap length constant to 100 s. In Fig. 8 the *D*_*gap*_ was set to 0.0001, to 0.01 and to 0.035 *μ*m^2^/s. The scatter plots show the resulting *D*_*gap*_ and *t*_*gap*_ for 100 different simulations. In the first case (*D*_*gap*_ = 0.0001 *μ*m^2^/s, Fig. 8 a, b) the transition is obvious. The Welch analysis yields reasonable results: in about 65% of the cases a gap with the right length is detected. The average *t*_*gap*_ is always overestimated, and therefore also the average *D*_*gap*_. The positional uncertainty also contributes to the overestimate of *D*_*gap*_, especially for low *D*_*gap*_ (see Additional file 1: Figure S4 a, b for *D*_*gap*_ = 0 and 0,001 *μ*m^2^/s). For this reason, only 10% of the cases yield *D*_*gap*_ within 20% of the input value.

In the second case, where *D*_*gap*_=0.01*μ*m^2^/s (Fig. 8 c, d), the transition is also clearly detectable. Both the correct *t*_*gap*_ and *D*_*gap*_ are detected in about 60% of the cases. In Fig. 8 d, it is clear that most incorrect values originate from a missed transition, which results in a double duration of the gap phase, and an increased *D*_*gap*_.

In the last case (Fig. 8 e, f), *D*_*gap*_ = 0.035 *μ*m^2^/s, only 30% lower than the *D* outside the gap. Given an uncertainty of at least 10% in the detection of the single diffusion coefficient (see previous paragraph), we expect this difference to be hard to detect. Indeed, looking at *D(t)* (Fig. 8 e) we can still distinguish a change in *D* in the common trend, but the fluctuations in each single curve obscure transitions in *D*. In less than 10% of the traces a gap with the correct length is detected, but the correct *D*_*gap*_ is detected in 60% of the cases. This is due to the small difference between *D* inside and outside the gap: in the cases where the transition is detected at a different point in time, the obtained *D* will still be good enough, being an average between *D* and *D*_*gap*_. In about 40% of the simulations no transition is detected (Fig. 8 f). In Additional file 1: Figure S3 more cases with different values of *D*_*gap*_ are reported.

Thus, changes in *D* smaller than 30% can easily be distinguished from averaged data, but in single trajectories a reduction of about 80% is required to detect 60% of such changes.

**Fig. 9:**
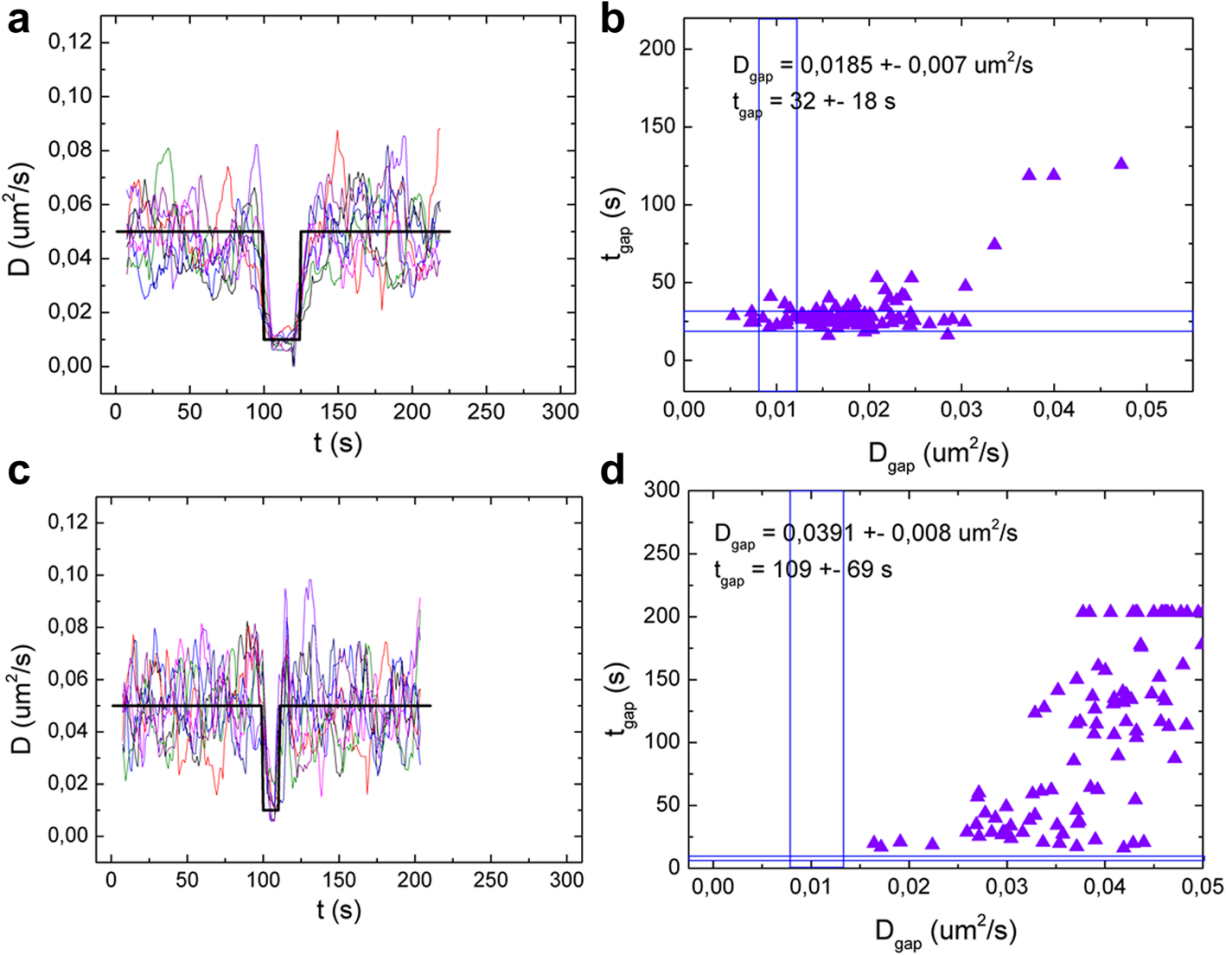
Limits to the detection of short-lived changes in diffusion. *D(t)* plots were obtained from rolling-window analysis (**a**, **c**) and the results of the MSD analysis are presented as scatter plots in **c** and **d**. The simulated *D*(t) is plotted in *black line*. The *D*_*gap*_ was 0.01 *μ*m^2^/s and the *D* outside the gap was 0,05 *μ*m^2^/s. *t*_*gap*_ = 25 s in **a**, **b** and *t*_*gap*_ = 10 s in **c**, **d**. The results can be compared to Fig. 8 (**c**, **d**) where the same *D*s were set with a gap length of 100 s. For every case 100 traces were simulated and analyzed: **a** and **c** show *D*(t) for 10 example traces; from all the traces are shown in **b** and **d**. The ranges of correct *D*_*gap*_ and *t*_*gap*_ are highlighted with *blue lines* in the scatter plot. The traces were analyzed using a rolling window size of 15 points, and a Welch sample of 15 points (**b**) and 10 points (**d**)

We expect transient changes to become more obscured as their duration shortens. We performed a similar analysis as function of the length of the gap *t*_*gap*_, keeping *D*_*gap*_ constant at 0.01 *μ*m^2^/s. In Fig. 9 the results obtained using a gap length of 25 s and 10 s are plotted. In the case of *t*_*gap*_ = 100 s, (Fig. 8 c, d), both the correct *t*_*gap*_ and *D*_*gap*_ are detected in about 60% of the cases. Reducing *t*_*gap*_ to 25 s (Fig. 9 a, b), in only 35% of the simulations the correct *t*_*gap*_ is detected, and the correct *D*_*gap*_ in 20% of the cases. A gap of only 10 s (Fig. 9 c, d) is very hard to detect: in none of the cases the correct *t*_*gap*_ or *D*_*gap*_ was detected. More results are reported in Additional file 1: Figure S4.

**Fig. 10:**
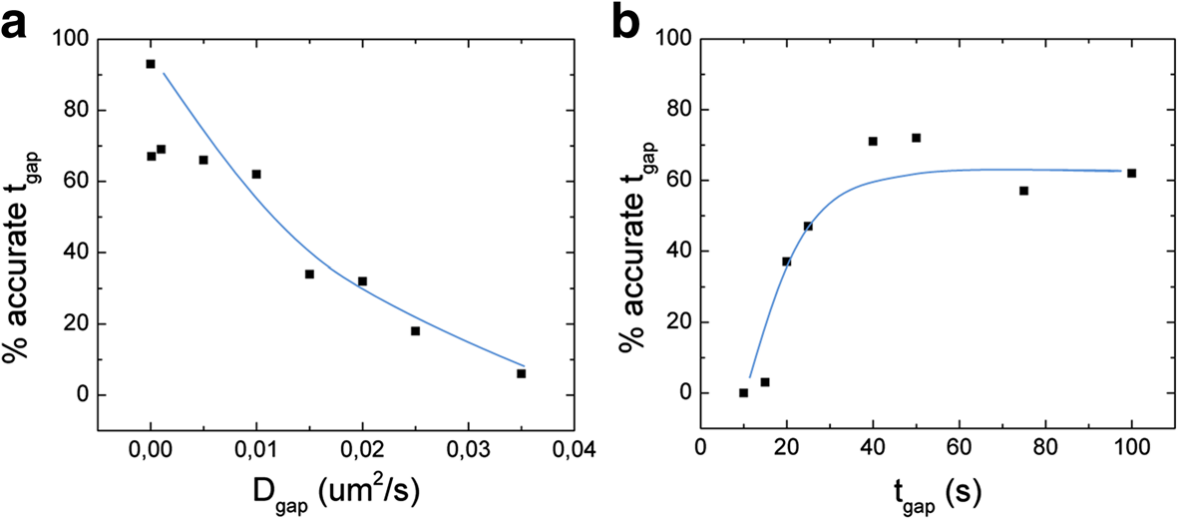
Percentage of accurate *D*_*gap*_ detections for different values of *D*_*gap*_ and *t*_*gap*_. The percentage of the gaps detected with a length within 10% of the input is shown, for different values of *D*_*gap*_ (**a**) and *t*_*gap*_ (**b**). Every point was obtained from 100 simulations of a trajectory containing a gap. The *D* outside the gap is 0,05 *μ*m^2^/s, *t*_*gap*_ = 100 s and *D*_*gap*_ = 0,01 *μ*m^2^/s, if not stated otherwise. *Lines* are a guide to the eye

It is difficult to give an absolute limit of gap detectability in terms of *D*_*gap*_ or *t*_*gap*_. A summary of the dependence of the detectability of the gap is plotted in Fig. 10. If the gap is long (100 s) and the ratio between *D* and *D*_*gap*_ is more than 50, at least 60% of the gaps are correctly assigned. If the length of the gap is reduced to 25 s we can still detect 50% of the gaps, but for gaps shorter than 20 s the detection rate drops to 0. We still detected a transition in 60% of the cases if *D*/ *D*_*gap*_=5. But for *D*/ *D*_*gap*_ =2, the gap was detected in only 20% of the cases, even for long traces (100 s). In conclusion, a transient decrease in *D* can be detected easily when the *D* in the gap is very low, and the length of the gap is not too short. A similar conclusion is obtained for the detectability of the correct *D*_*gap*_, with a difference: a small *D*_*gap*_ won’t be fit correctly due to the noise introduced by the positional uncertainty (Additional file 1: Figure S5).

To improve the precision of the results, a more complex measurement and analysis scheme could be used, that makes use of more parameters to detect subsections; for example, one could simultaneously measure the polarization of the signal. When applicable, other mobility parameters such as direction of the motion, velocity or confinement could be fit to the MSD curves. When such parameters are different during the binding of GNRs to cellular structure, Welch analysis can be performed, as in [28, 29]. The final *p*-value, obtained by multiplying the *p*-values of different parameters, will give a more correct assignement of the transition points and consequently more precise estimates of *t*_*gap*_ and *D*_*gap*_.

In practice, measuring *D* and *D*_*gap*_ of a protein will depend on the size of the protein and on the local viscosity of the environment. The smaller the molecules, the larger the difference in the diffusion coefficient when it binds to its substrate and the easier to detect the event accurately. The size of the GNR will set an upper limit to the diffusion coefficient that will be measured. The affinity of the protein is directly reflected in the ratio of the time between binding events and the lifetime of the bound complex, the latter being referred to here as *t*_*gap*_. The first may be affected by the presence of a GNR. A wide range of binding times have been reported for example for DNA binding proteins in vivo, ranging from sub-seconds [26] to several minutes [30]. The ability to track a single protein bound to a GNR will give a more detailed insight in the reaction kinetics and how the complex cellular environment affects this reaction. Here we have shown that using GNRs as labels can, in many conditions, resolve single binding events with nanometer and second accuracy.

## Conclusion

Quantification of diffusion is challenging, especially under experimental conditions with limited accuracy, time resolution and finite length of the measurement. By performing simulations and experiments in controlled conditions, we established few guidelines to minimize the error on the MSD and consequently on *D*:

1. use long trajectories: the larger the number of time points in the trace the better the MSD is; in our case, doubling the trace length from 1 min to 2 min yielded a two-fold improvement of the precision in the detected *D*.
2. in case of small positional uncertainties, theory and results from simulations suggest to use only the first two points for fitting the MSD.
3. fixing the positional uncertainty during the MSD fit improves the evaluation of *D*.

These findings reinforce previous theoretical reports [8, 13]. In our case we tracked GNRs in 3D with an uncertainty of 4 nm based on shot-noise limitations, which increased to about 40 nm due to the movement of particles between each acquisition. With these conditions, the best approximation of *D* was within 10% of the expected value of *D*. Such a high precision could not be achieved using fluorophores as GFP or synthetic dyes as quantum dots, because their low signal provides a low spatial resolution, and their bleaching or blinking behavior make it impossible to collect long trajectories.

Given the challenges to extract a precise value of the diffusion coefficient, the analysis of changes in mobility needs extra care. We simulated traces with ‘gaps’ in the diffusion, as it can occur when a particle is temporarily immobilized, for example by specific binding to a cellular structure. The detectability of such gaps depends critically on the difference in the diffusion before and during the binding, determined mainly by the size of the ligands, and the length of the binding event. In our conditions and optimizing the MSD analysis as described, the detection of the gap was possible with a probability equal or higher than 50% only when the gap was longer than 20 s and the *D* in the gap was less than 5 times smaller than the *D* in the rest of the trace. These findings are applicable for all types of SPT methods in which individual traces are analyzed without averaging. We expect that many events characterized by a short duration or inducing a limited change in diffusion are overlooked in such experiments because of the stochastic character of diffusion. In any case, using large particles may produce brighter and more stable signals, but reduces the diffusion coefficient, making the difference in *D* between free and immobile particles smaller.

## Note added in Proof

After the manuscript was accepted, we learned that Mortensen et al. (Optimized localization analysis for single-molecule tracking and superresolution microscopy, 2010, Nature Methods) pointed out that Eq. 1 overestimates the 2D positional accuracy by 30%, which agrees very well with the 2D simulation results, as shown in Fig. 3 a. For 3D tracking, this correction is counterweighted by the improved accuracy resulting from fitting multiple slices, also shown in Fig. 3 a. This empirical observation validates using Eq. 1 for estimations of the offset in the MSD plots.

## Additional file

Additional file 1 Supplementary Figures. The PDF files contain supplementary figures and a supplementary table. (PDF 1403 kb)

## Abbreviations

FCS: Fluorescence Correlation Spectroscopy
GNR: Gold nanorod
MSD: Mean Square Displacement
PEG: Polyethilene-glycol
SMT: Single Molecule Tracking
SPT: Single Particle Tracking
TEM: Transmission electron microscope
