# Supplementary material for: Accuracy of the detection of binding events using 3D single particle tracking
Source: BMC Biophys. 2017 Mar 23;10:3. doi: 10.1186/s13628-017-0035-8 (PMC5364544; doi:10.1186/s13628-017-0035-8)
Supplement: Additional file 1 — Supplementary Figures. The PDF files contain supplementary figures and a supplementary table. (PDF 1403 kb) [file 13628_2017_35_MOESM1_ESM.pdf]

Supplementary Figures

Suppl. Fig. 1. Fluorescence Correlation Spectroscopy of GNRs in water to determine the diffusion coefficient. A solution of 47 nm x 14 nm GNRs in water was used for the experiment. The fit gave τ_D = 3,85 10^{-3} s. From τ_D a D = 7.5 10^{-8} m^2/s is calculated.

The particle radius is calculated from *D* using Eq. 3, yielding a value of 29,1 nm. In the case of non-spherical particle like GNRs, an 'equivalent radius' (radius of a sphere having an equivalent volume) must be used in Eq. 3. In case of rods, the equivalent radius is defined as:

$$r = \sqrt[3]{ml^2}$$

where *m* and *l* are the longer and shorter axis. For 47 nm x 10 nm GNRs, the equivalent radius is equal to 21 nm. Subtracting this value from the radius obtained from the FCS, a radius of 8,1 nm was obtained for the PEG layer. The FCS experiment was performed on the setup described in Koopmans et al, 2009 (*spFRET Using Alternating Excitation and FCS Reveals Progressive DNA Unwrapping in Nucleosomes*, Biophysical Journal), using 100-nm tetraspeck fluorescent beads (Invitrogen) to calibrate the focal volume.

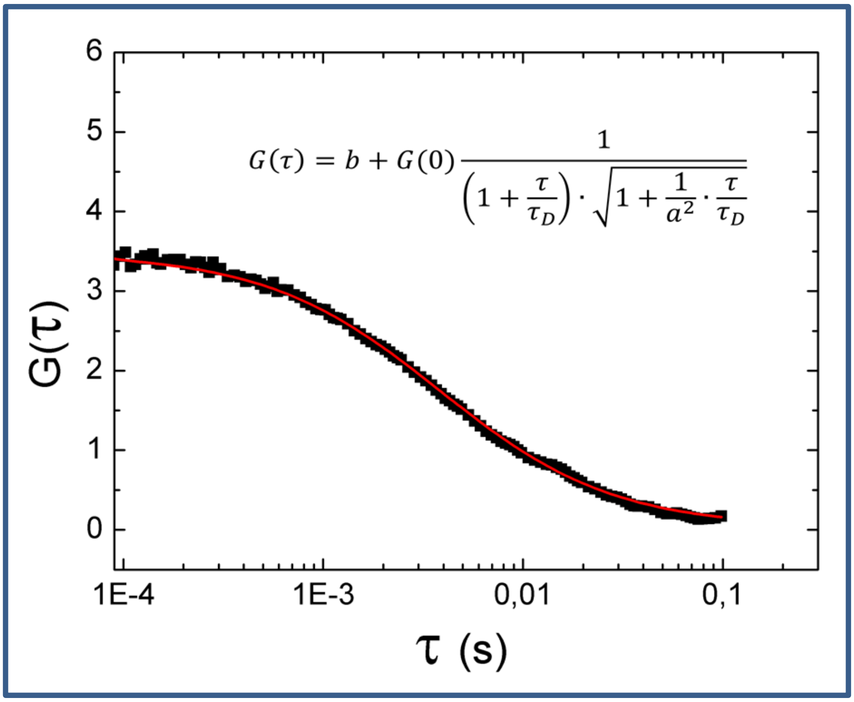

Supplementary fig. 2. The large number of photons emitted by GNRs gives a high positional accuracy. The distribution of the number of collected photons N_p (**a**) and relative positional accuracies σ (**b**)obtained in our setup are shown. Samples of 47 nm x 14 nm and 53 nm x 16 nm GNRs in 95% glycerol were used. The values of σ were obtained from *Np* using Eq. 1. The width of the distribution of collected photons (and thus of the distribution of positional accuracies) reflects the size dispersion of the particles within the sample.

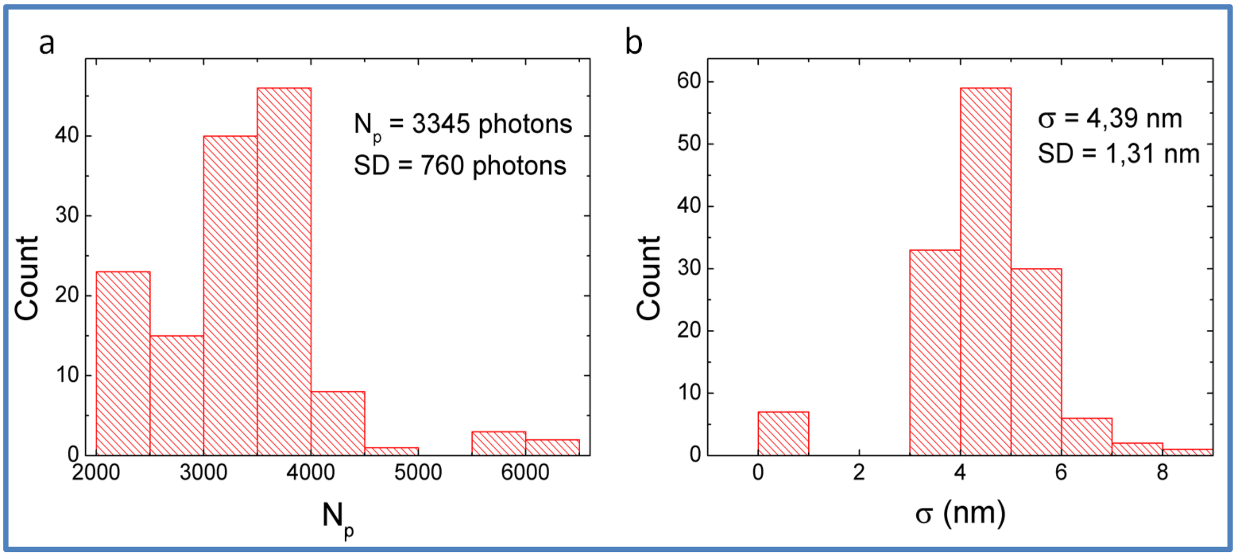

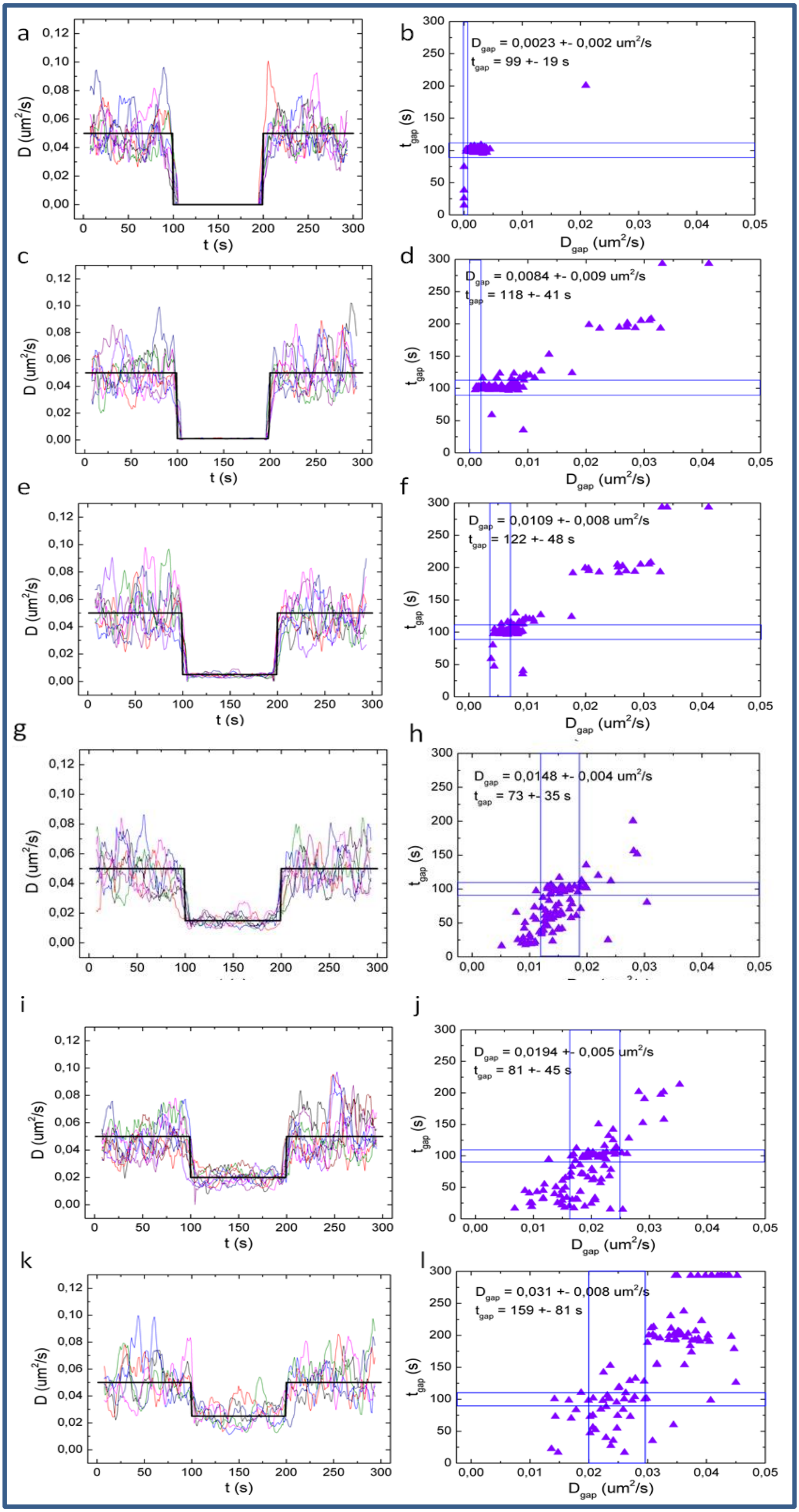

Suppl Fig. 3. Detectability of changes in mobility in simulated traces with different *Dgap*. The *D*(t) plots obtained from the rolling-window analysis (**a,c,e,g,i,k**) and cloud plots containing *tgap* vs *Dgap* (**b,d,f,h,j,l**) are shown. The simulated *D*(t) (black line) is overlapped to the detected *D* plots (color lines). The *D* outside the gap was 0.05 um2/s; while *Dgap* = 0 um^2/s in **a,b**, *Dgap* = 0,001 um^2/s in **c,d**, *Dgap* = 0,005 in **e,f**, *Dgap* = 0,015 um^2/s in **g,h**, *Dgap* = 0,020 um^2/s in **i,j**, and *Dgap* = 0,025 um^2/s in **k,l**.
The ranges of correct *Dgap* and *tgap* are highlighted with blue lines in the scatter plot. The trace were analyzed using a rolling window size of 15 s and a Welch sample of 15 points.

Suppl Fig. 4. Detectability of changes in mobility in simulated traces for different durations of the gap. The *D*(t) plots obtained from the rolling-window analysis (**a,c,e,g,i**) and plots containing *D* in function of the length of the detected gaps (**b,d,f,h,l**) are shown for more *D* tested. The simulated *D*(t) (black line) is overlapped to the detected *D* plots (color lines). The *D* outside the gap was 0.05 um2/s and *Dgap* is 0,01 um2/s. *tgap* = 75 s in **a,b**, *tgap* = 50 s in **c,d**, *tgap* = 40 s in **e,f**, *tgap* = 25 in **g,h** and *tgap* = 20 s in **i,l**. The ranges of correct *Dgap* and *tgap* are highlighted with blue lines in the scatter plot. The traces were analyzed using a rolling window size of 15 s in all three cases, and a Welch sample of 15 points for the first four cases, and 10 points for the last case.

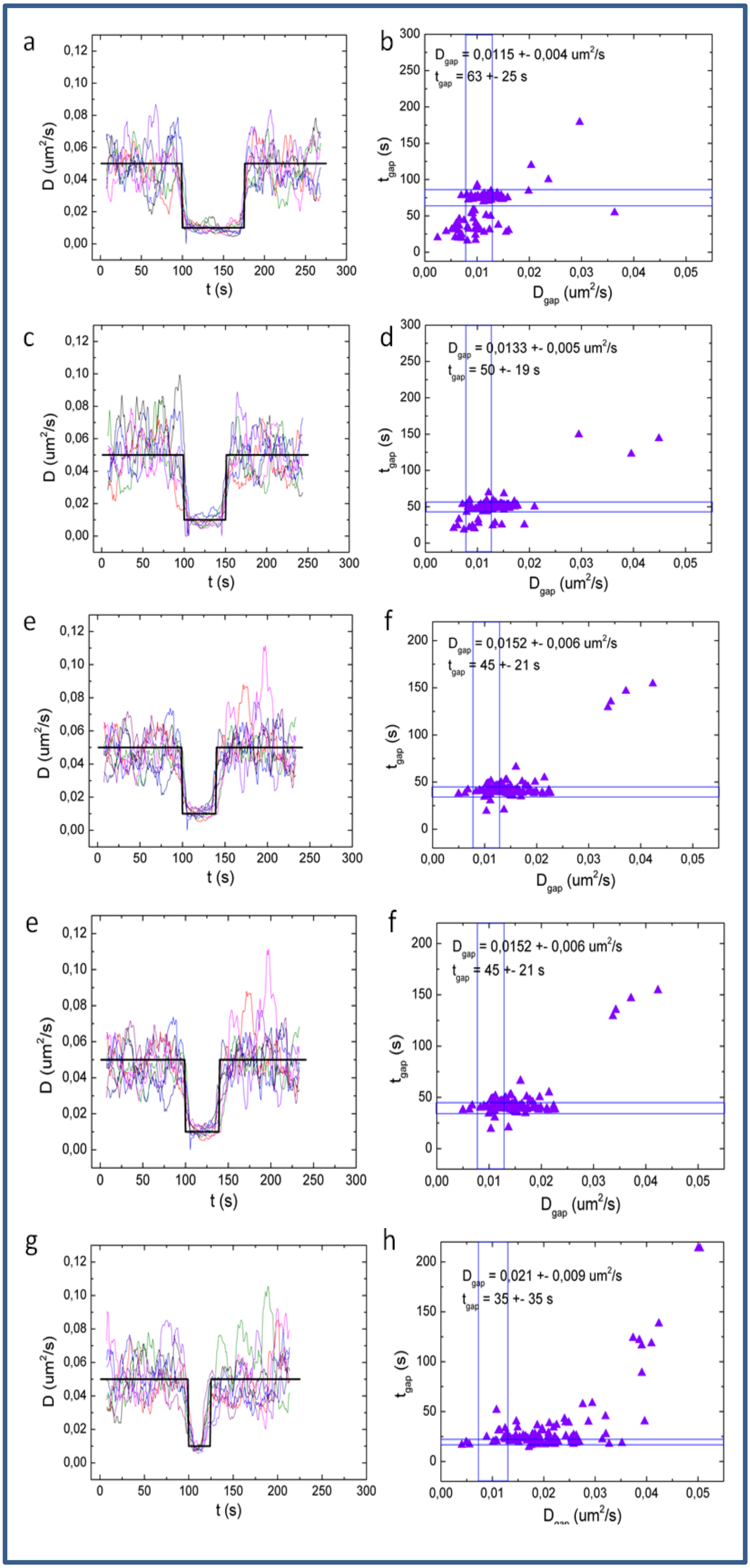

Suppl. Fig. 5 Percentage of detections of accurate *Dgap* for different values of *Dgap* and *tgap*. The percentage of *Dgap* within 20% of the input is shown, for different values of *Dgap* (**a**) and *tgap* (**b**). Every point is obtained from 100 simulations of the trajectory containing a gap. The *D* outside the gap is 0,05 um^2/s, *tgap* = 100 s and *Dgap* = 0,01 um^2/s, if not stated otherwise. Lines are a guide to the eye.

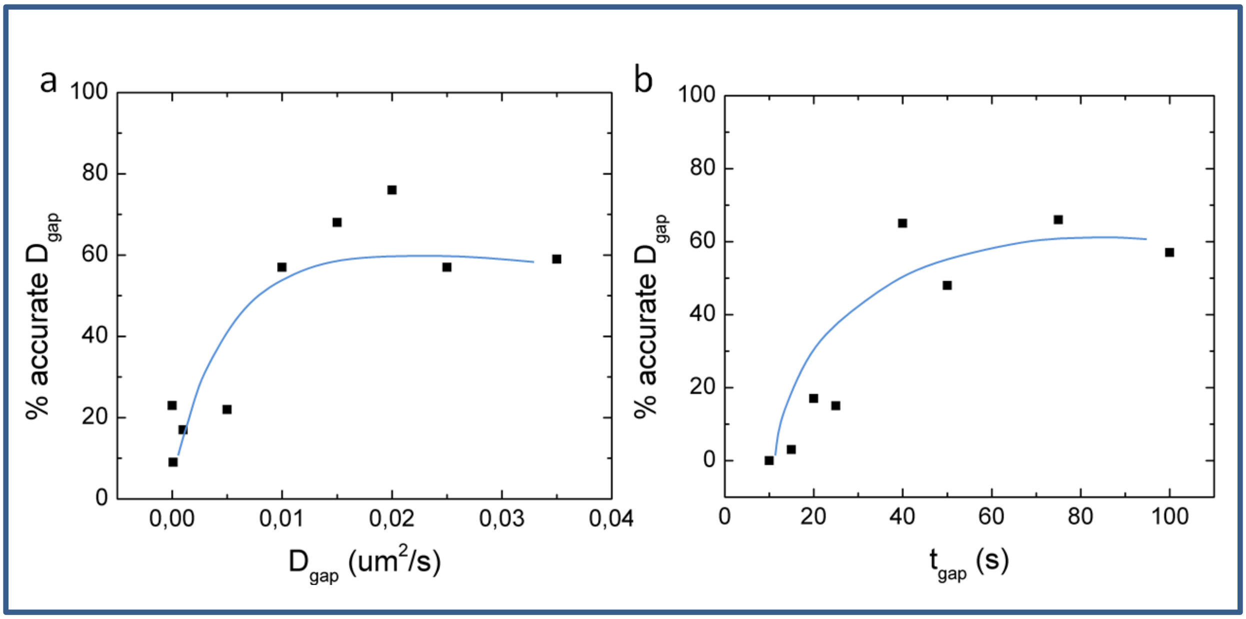

sample	expected *D* (um^2/s)	measured *D* (um^2/s)		
		all traces	traces > 40 points	traces > 80 points
47nmx14nm GNRs 90% glycerol	0,065 ± 0,003	0,04 ± 0,02	0,043 ± 0,01	0,05 ± 0,01
47nmx14nm GNRs 95% glycerol	0,03 ± 0,001	0,02 ± 0,01	0,022 ± 0,005	0,025 ± 0,003
53nmx16nm GNRs 95% glycerol	0,028 ± 0,003	0,02 ± 0,01	0,022 ± 0,006	0,026 ± 0,005

Supplementary table 1. Comparison of the diffusion coefficient *D* as calculated using the Stokes-Einstein relation with experimental data. The expected values were calculated using Eq. 3, assuming a temperature of 25 degrees and using an 'equivalent radius' as described in Supplementary fig. 1. The expected variations in *D* were calculated according to the size dispersion within the sample. The table reports values of experimental *D* obtained using all the traces, or only traces longer than 40 points and 80 points.
